# Supplementary figures and images for: Cryo-EM structure of the endothelin-1-ETB-Gi complex
Source: eLife. 2023 Apr 25;12:e85821. doi: 10.7554/eLife.85821 (PMC10129325; doi:10.7554/eLife.85821)

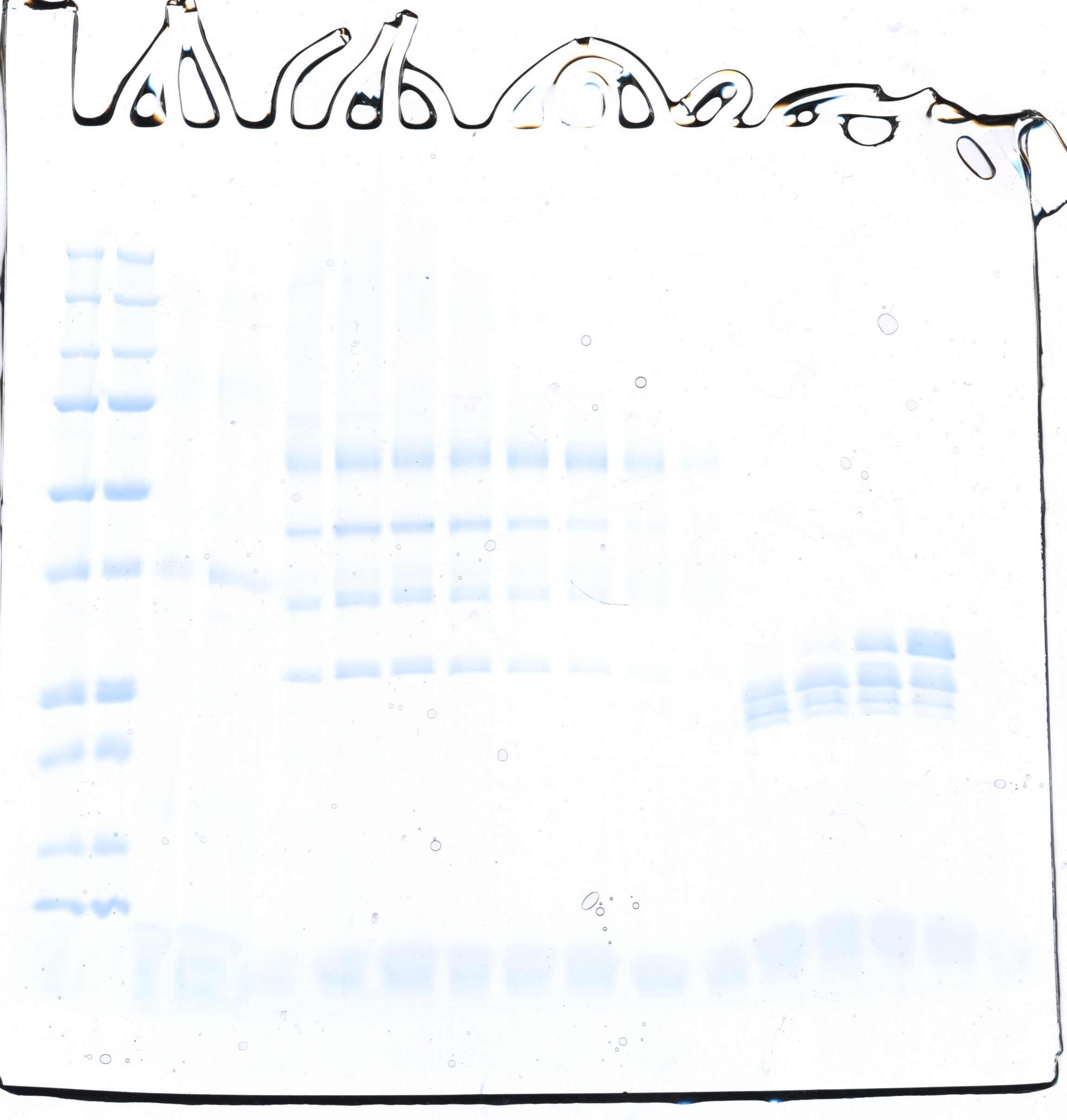

Supplement: Figure 1—figure supplement 1—source data 1. [file elife-85821-fig1-figsupp1-data1.zip › Nureki_24-12-2022-SR-eLife-85821R1_Related_Manuscript_File (3).jpg]
